# T156. IN VIVO CHARACTERIZATION OF THE FIRST AGONIST DOPAMINE D1 RECEPTORS PET IMAGING TRACER [18F]MNI-968 IN HUMAN

**DOI:** 10.1093/schbul/sby016.432

**Published:** 2018-04-01

**Authors:** Gilles Tamagnan, Olivier Barret, David Alagille, Vincent Carroll, Jennifer Madonia, Cristian Constantinescu, Christine SanDiego, Caroline Papin, Thomas Morley, David Russell, Timothy McCarthy, Lei Zhang, David Gray, Anna Villalobos, Chewah Lee, Jianqing Chen, John Seibyl, Kenneth Marek

**Affiliations:** 1 inviCRO; 2 Constantinescu; 3 Pfizer, Inc

## Abstract

**Background:**

D1 receptors, which couple to inhibitory G-proteins, have been shown to regulate neuronal growth and development, mediate some behavioral responses. Its function has been shown to be altered in both neurologic and psychiatric disorders. To date, there is a lack of agonist PET tracers for the D1 receptors labeled with 18F with relevance in clinical studies. We report the evaluation in non-human primates of [18F]MNI-968 (PF-06730110), a novel PET radiotracer of the D1 receptors

**Methods:**

Four brain PET studies, 2 baselines and 2 blockade studies using PF-2562, a D1 partial agonist compound, were conducted for 90 min in two rhesus monkeys with [18F]MNI-968 (169 ± 31 MBq). [18F]PF-06730110 was administered at the same dose level for both monkeys as a bolus followed by a 2-hour infusion, with [18F]MNI-968 administered 30 min into the infusion. Additionally, six brain PET studies were conducted over 180 min (317 ± 49 MBq) in 6 healthy human volunteers (3 test/retest and 3 test). PET data were modeled with 2-tissue compartmental model (2T), Logan graphical analysis (LGA), and non-invasive Logan graphical analysis (NI-LGA) with cerebellar cortex as reference region to estimate total distribution volume VT, and binding potential BPND.

For the blockade studies in rhesus monkeys, occupancy was estimated from BPND at baseline and post blockade.

**Results:**

In rhesus monkeys, [18F]MNI-968 (PF-06730110), penetrated the brain with a peak whole-brain uptake up to ~3% of the injected dose at ~ 6 min post injection and showed a fast washout. The highest signal was found in the caudate, putamen, with moderate extrastriatal uptake. The lowest signal was in the cerebellum. BPND values were up to ~1.4 in the putamen. All three quantification methods (2T, LGA and NI-LGA) were in excellent agreement, with a similar estimated D1 receptors occupancy of PF-06730110 of ~40% for both monkeys in the caudate and putamen.

In human, [18F]MNI-968 kinetics appeared to be faster compared to non-human primates, with a BPND in the putamen of ~0.8. Initial measurement of test-retest reproducibility was ≤ 7% for BPND in the striatal regions.

**Discussion:**

Our work showed that [18F]MNI-968 ([18F]PF-06730110), is a promising agonist PET radiotracer for imaging D1agnist receptors that can be quantified non-invasively. Studies are currently ongoing both in non-human and human primates to further characterize the tracer.

